# Explainable artificial intelligence driven insights into smoking prediction using machine learning and clinical parameters

**DOI:** 10.1038/s41598-025-09409-w

**Published:** 2025-07-05

**Authors:** S. Aishwarya, P. C. Siddalingaswamy, Krishnaraj Chadaga

**Affiliations:** Manipal Institute of Technology, Manipal Academy of Higher Education, Manipal, India

**Keywords:** Smokers detection, Machine learning, Artificial intelligence, XAI, Health parameters, Risk factors, Epidemiology

## Abstract

Smoking is a leading cause of various health conditions, including cancer and respiratory diseases. Smokers often face medical restrictions such as limitations in blood and organ donation, reduced effectiveness of medications, and increased surgical complications. These impacts underscore the need for early detection of smoking status to enable timely intervention. This study explores the use of Artificial Intelligence (AI) and Machine Learning (ML) techniques to predict smoking status based on health parameters, including biosignals and clinical biomarkers. A balanced subset of 2,000 instances was sampled from a publicly available Kaggle dataset comprising clinical and biometric features. Multiple ML models were implemented, including Random Forest Classifier, Logistic Regression, Decision Tree Classifier, K-Nearest Neighbors, CatBoost Classifier, and an Artificial Neural Network. The Random Forest Classifier achieved the better performance with an accuracy of 0.80, precision of 0.80, recall of 0.80, and F1-score of 0.79. To enhance model interpretability, four Explainable Artificial Intelligence (XAI) techniques were applied: Shapley Additive Explanations (SHAP), Local Interpretable Model-Agnostic Explanations (LIME), QLattice, and Anchor. SHAP identified hemoglobin as the most influential predictor, while LIME, QLattice, and Anchor highlighted the role of gamma-glutamyl transferase (t). Interactions between hemoglobin, GTP, and height were associated with more accurate predictions. The integration of ensemble modeling and multiple XAI approaches offers deeper interpretability than prior studies, providing healthcare providers and policymakers with a robust, transparent decision-support tool for targeted intervention strategies.

## Introduction

Smoking is a primary cause of preventable diseases and deaths around the world. It increases the possibility of getting life-threatening diseases like cancer, cardiovascular disease, chronic obstructive pulmonary disease (COPD), and respiratory infections^1^. Smoking impairs the immune system, leaving people more prone to infections and slowing their recovery from illnesses^2^. Furthermore, smokers are more likely to face medical constraints such as ineligibility for blood donation, reduced drug effectiveness, an increased risk of surgical complications, and exclusion from organ donation eligibility^3^. The widespread health and societal consequences of smoking highlight the critical need for effective prevention and cessation strategies^4^.

**Fig. 1:**
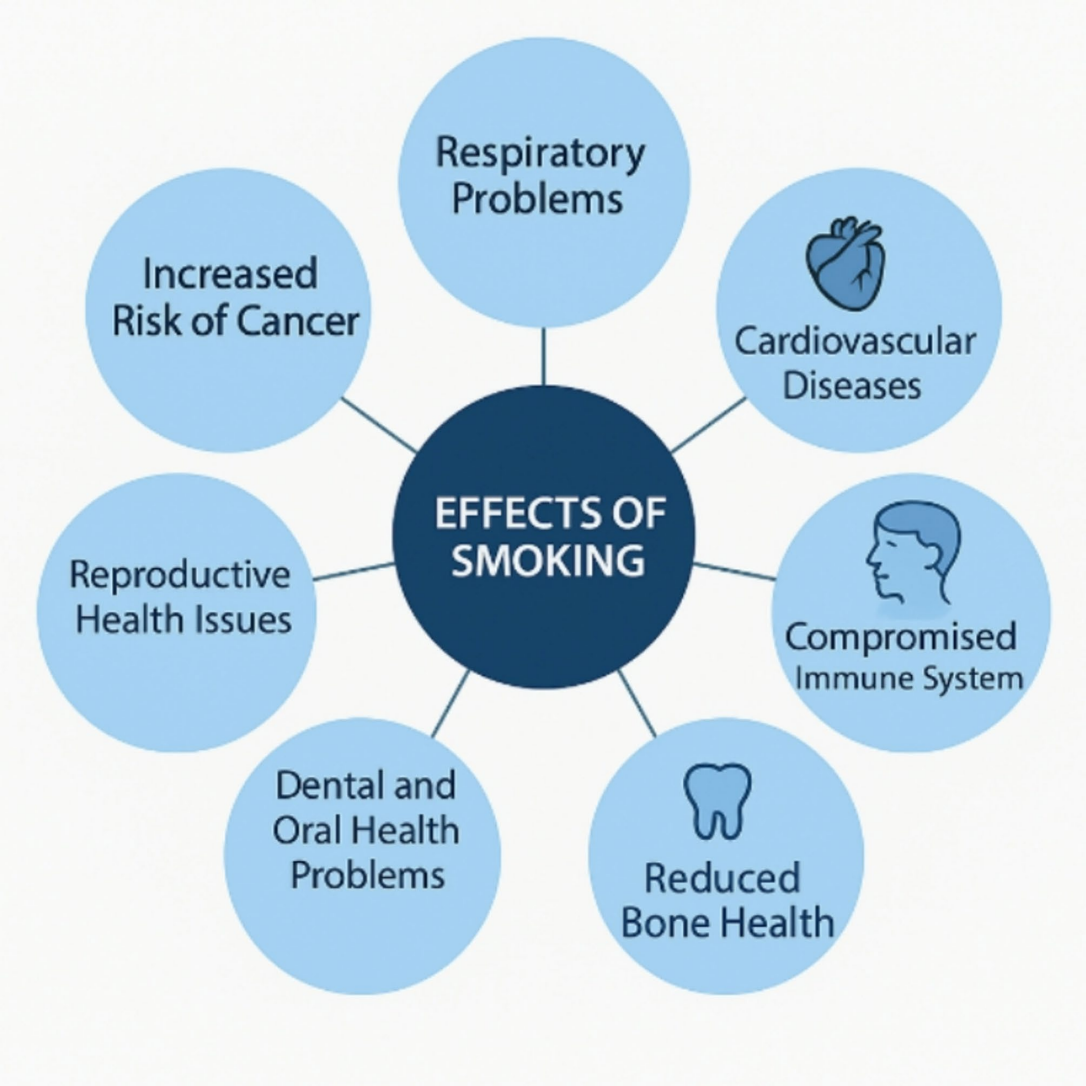
Health effects attributed to smoking.

Early diagnosis of smoking habits is critical for timely medical intervention and the avoidance of serious health consequences^5^. Many people, however, choose to conceal their smoking habits for a variety of reasons, including social stigma, fear of judgment, personal embarrassment, and concerns about employment consequences^6^. This hiding hinders effective medical diagnosis and delays necessary measures, increasing medical risks over time^7^. Detecting smoking status via non-invasive, objective approaches becomes critical in these circumstances, allowing healthcare practitioners to intervene without relying exclusively on self-reported data, which is frequently incorrect^8^. Figure 1 denotes the common effects caused by smoking.

Artificial Intelligence (AI) and Machine Learning (ML) are gaining prominence as transformative instruments in healthcare, providing answers to challenging problems like predicting smoking patterns^9^. AI and ML algorithms detect whether someone smokes by assessing various health factors such as biosignals, clinical measures, and demographic data^10^. They are accurate and scalable. However, the lack of transparency of conventional AI systems frequently limits their applicability in sectors such as healthcare^11^. Explainable AI (XAI) fills the gap by providing insights into model decisions and ensuring openness. By highlighting the importance of attributes, XAI models assist medical personnel in accepting AI-driven recommendations and interpreting predictions^12^. These developments demonstrate how AI and XAI can create reliable and understandable systems for early smoking detection and tailored therapies.

.

A few studies exist where AI has been used to predict whether a person smokes or not. Ammar et al.^13^ used Kaggle bio signals data (304,411 cases, 43 characteristics) to predict smoking status. After preprocessing the data with strong scaling, using Random Forest for feature selection, and applying Borderline-SMOTE for class balancing, they developed the Proposed Blending Model (PBM), which combines Echo State Network, GoogleNet, and AlexNet. The model outperformed baselines by 5–7% in accuracy (77%), precision (72%), recall, and F1-score. SHAP explained model projections, and 10-fold cross-validation confirmed its reliability, highlighting its potential for early cardiovascular health intervention.

Singh and Mantri^14^ proposed a hybrid approach combining Rough Set Theory (RST) and machine learning algorithms to predict smoking status from health-related features. They employed RST for attribute reduction, followed by classifiers such as Decision Trees, Naive Bayes, and Support Vector Machines. Their method improved prediction accuracy while reducing computational complexity. Feature significance and classification performance were analyzed using metrics like accuracy and sensitivity.

Singh and Mantri^15^ proposed a clinical decision support system combining rough set theory and machine learning to predict diseases like hepatitis, dermatological conditions, hepatic disease, and autism. Using rough set-based feature selection and classifiers such as random forest, their model achieved high accuracy—up to 100% for autism detection. Performance was evaluated with precision, recall, F1-score, and RMSE.

Singh and Kumar^16^ developed a hybrid AR and RST system for disease prediction from incomplete symptom data, achieving high accuracy in neurodevelopmental disorders. RST effectively manages data uncertainty, while preprocessing techniques improve model robustness. Other studies demonstrate rule-based methods’ effectiveness in various medical diagnoses, especially with noisy or incomplete data.

McCormick et al.^17^ used semantic cues to classify patient smoking status. The dataset used was the i2b2 dataset of medical summary of discharge, which was used to determine smoking status. They evaluated MedLEE, SVM, and KNN to Okapi-BM25, Naïve Bayes, and BoosTexter classifiers. The MedLEE-based classifier performed the best, with an F-measure of 0.89, whereas the rule-based classifier had an F-measure of 0.83.

Singh et al.^18^ developed a Clinical Decision Support System using logistic regression and clustering to predict patient diagnosis urgency and applied churn analysis to identify patients likely to discontinue care. Their model achieved 74% accuracy and showed improved decision-making and healthcare efficiency.

Ahmed et al.^19^ used SPSS and the Urite 3000 plus analyzer to investigate the impact of tobacco smoking on hematological markers in Sudanese male smokers. Smokers had more significant RBC, Hb, WBC, and neutrophil counts than non-smokers. The study underscored the importance of individualized smoking cessation treatments.

Badicu et al.^20^ predicted tobacco and alcohol intake among Romanian students based on demographic characteristics and physical activity level using a dataset of 253 participants. Pearson’s correlation and ordinal logistic regression were among the statistical methods used. The study discovered high rates of problematic alcohol use, moderate tobacco usage, and a negative relationship between physical activity and substance use.

Münzel et al.^21^ evaluated the effects of tobacco, e-cigarettes, and waterpipe smoking on human endothelial function and found that all types damage vascular health, increasing oxidative stress and inflammation. They used flow-mediated dilation (FMD) and biochemical assays to evaluate oxidative stress markers and inflammatory responses, as well as imaging and molecular biology techniques.

Groenhof et al.^22^ developed a rule-based data mining algorithm integrating structured and unstructured EHR data, achieving high sensitivity (88%) and specificity (92%) in identifying current smoking status. They utilized natural language processing (NLP) techniques and clinical decision rules, but limitations include potential misclassification due to conflicting data sources and reliance on clinician-documented information. Further studies employing machine learning approaches like word2vec and deep learning show promise for improved accuracy in smoking status detection.

Fan and Gao^23^ proposed a wearable system utilizing motion sensors from smartphones and smartwatches, employing a hybrid variational autoencoder and neural decision forest to detect smoking events with high accuracy (96.29% F1-score). They validated the model on a large dataset, demonstrating efficiency and robustness. However, the approach may face limitations in real-world scenarios with diverse activities and postures that could affect sensor data consistency.

Ton That et al.^24^ used a dataset of 55,693 instances and applied LASSO for feature selection followed by ML classifiers—Random Forest, XGBoost, LightGBM, and MLP—to predict smoking status. Random Forest achieved the best accuracy of 84.73% with improved F1-score and precision. While performance improved with LASSO, limitations included lack of advanced imputation techniques and imbalance handling, suggesting future use of SMOTE and other feature selectors.

de Luna et al.^25^ used machine learning, notably Random Forest, to classify smokers and non-smokers based on 17 health features, achieving 88.03% training and 83.29% testing accuracy. The model was deployed on a Raspberry Pi with a touchscreen. While effective, the system’s limitations include minimal accuracy gains from tuning and a small, less user-friendly display.

Thakur et al.^26^ utilized machine learning models, including Random Forest and XGBoost, trained on sensor, blood test, and lifestyle data to predict smoking and drinking behaviours, achieving up to 79.65% and 73.96% accuracy respectively. They incorporated explainability tools like SHAP and LIME to interpret model predictions, but the reliance on specific biological datasets may limit real-world scalability. Table 1 lists studies that do the smoking prediction.

**Table 1:** Literature that uses AI and ML for smoking prediction.

Reference	Dataset	Model used	Result	Novelty
^22^	19,410 Instances	classification and regression trees	80% accuracy	-
^23^	9 Instances	Neural Decision Forest (VARST) and a Variational Autoencoder (VAE)	96.29%. F1-score	-
^24^	55,693 Instances	Various supervised models.	84.73% accuracy	Implements LASSO for dimensionality reduction
^25^	55,692 Instances	Various supervised models.	83.29% accuracy	-
^26^	991,346 Instances	Various supervised models.	79.65% accuracy	Model transparency through XAI

Existing research focuses on distinct machine learning models, with little investigation into heterogeneous or hybrid approaches that integrate different AI/ML techniques. Statistical examination of feature significance and interactions is frequently ignored, resulting in gaps in our understanding of the links between health metrics and smoking status. Many studies did not explain the reasoning behind the smoking prediction. Although previous studies have applied ML and DL techniques to smoking status prediction, many lacked transparency, explainability, or focused solely on classification accuracy. Few incorporated diverse XAI methods or investigated feature interactions in depth. This study addresses these gaps by integrating multiple interpretable AI models and statistical validation techniques to offer both predictive accuracy and insight into contributing health factors.

This study seeks to address the identified research gaps, with its key contributions outlined as follows:

- Extensive statistical evaluation is done using Jamovi. where various descriptive and inferential statistical techniques are applied to analyze the dataset.
- Heterogenous XAI methods are made use of to facilitate a better understanding of machine learning models.
- Various machine learning models, including a custom ensembling technique, are used to ensure good performance and effective evaluation.
- The essential parameters were compared using statistical techniques, mutual information, Pearson’s correlation, and several XAI methods.
- Focus on the Generalizability and Real-World Applicability of our smoking prediction classifier.

The remainder of the paper is organized as follows: The following section describes the dataset and preprocessing steps. The methodology and machine learning models are then presented, followed by a discussion of evaluation metrics and experimental results. Subsequently, the explainable AI (XAI) techniques applied in the study are discussed. The final sections provide a discussion of the findings, limitations, and practical implications, and conclude with future research directions. The table x lists the acronyms used in this paper.

Table 2 tells the Acronym used throughout this paper.

**Table 2:** List of acronym used in the paper.

Acronym	Full Form
AI	Artificial Intelligence
ML	Machine Learning
XAI	Explainable Artificial Intelligence
SHAP	Shapley Additive Explanations
LIME	Local Interpretable Model-Agnostic Explanations
ANN	Artificial Neural Network
AUC	Area Under the Curve
AP	Average Precision
JS	Jaccard Score
LL	Log Loss
MCC	Matthews Correlation Coefficient
GTP	Gamma-glutamyl Transferase
Hb / HGB	Hemoglobin
IQR	Interquartile Range
KNN	K-Nearest Neighbors
ALT	Alanine Aminotransferase
AST	Aspartate Aminotransferase
LDL	Low-Density Lipoprotein
HDL	High-Density Lipoprotein
SPSS	Statistical Package for the Social Sciences
SMOTE	Synthetic Minority Over-sampling Technique
PBM	Proposed Blending Model
ROC	Receiver Operating Characteristic
TP/TN/FP/FN	True/False Positives/Negatives

## Materials and methods

### A. Data description

The dataset required for the study is available on Kaggle and was uploaded by Gaurav Dutta^27^. It contains 23 attributes, including five categorical and 18 continuous attributes. The target variable is Smoking, with values of ‘1’ for smokers and ‘0’ for non-smokers. Most categorical features have two values (e.g., 1 = normal, 2 = impaired for hearing), while urine protein has six levels. The continuous variables have different value ranges—for example, height ranges from 135 to 190 cm, weight from 30 to 125 kg, and systolic blood pressure from 79 to 240 mmHg. Table 3provides a brief overview of the attributes recorded in the dataset. The dataset comprises a total of 38,984 instances. Practical constraints necessitated sampling the dataset^28^. Sampling has numerous benefits, including cost and time efficiency, flexibility for learning huge populations, and the capacity to collect accurate, reliable insights from a representative fraction, allowing for focused analysis and reducing resource needs. In our study, sampling was performed to extract 2,000 data instances without replacement. The sample size was chosen to ensure a representative distribution of both groups while keeping the computational requirements manageable. A subset of 2,000 instances allows for efficient processing while maintaining sufficient variability in the data to train and evaluate the models effectively. Among these, 1000 were positive (smokers) and 1000 were negative (non-smokers), allowing for an equal class distribution suitable for classification tasks. Randomization was performed during the sampling phase to ensure an unbiased representation of smokers and non-smokers^29^. A probability sampling specifically stratified random sampling method was used to maintain balance across the target classes (smokers vs. non-smokers). Blinding was not applicable since this is a secondary dataset analysis^30^. Notably, the dataset did not contain any null values.

**Table 3:** Overview and description of the dataset used in the Study.

Attributes	Description
Age	The individual’s age classified into 5-year ranges to represent their age group.
Height(cm)	The individual’s height is recorded in centimeters
Weight(kg)	The individual’s weight is recorded in centimeters
Waist(cm)	The individual’s waist measurement, expressed in centimeters
Eyesight(left)	Visual acuity of the left eye usually expressed as a decimal value
Eyesight(right)	Visual acuity of the right eye usually expressed as a decimal value
Hearing(left)	Hearing ability of the left ear, usually categorized
Hearing(right)	Hearing ability of the right ear, usually categorized
Systolic Blood pressure	The systolic blood pressure represents the pressure in the arteries during the heart muscle contraction.
Relaxation Blood pressure	The diastolic blood pressure represents the pressure in the arteries when the heart muscle rests between beats.
Fasting blood sugar	Blood sugar levels are measured after fasting for a specific duration, indicating glucose levels.
Cholesterol	The total cholesterol level in the blood is used to assess cardiovascular health.
Triglyceride	The level of triglycerides (a type of fat) in the blood.
HDL	High-density lipoprotein cholesterol is known as “good cholesterol”.
LDL	Low-density lipoprotein cholesterol, often referred to as “bad cholesterol.”
Haemoglobin	The concentration of hemoglobin in the blood.
Urine protein	The presence of protein in the urine.
Serum creatinine	A measure of creatinine levels in the blood.
AST	Aspartate aminotransferase is an enzyme found in the liver and other tissues.
ALT	Alanine aminotransferase is another enzyme associated with liver health.
GTP	Gamma-glutamyl transferase, an enzyme indicating liver function and alcohol consumption levels.
Dental caries	The presence of cavities or tooth decay
Smoking	Smoking status of the individual

### B. Statistical analysis

Data analysis was conducted using Jamovi for statistical analysis^31^. For computational efficiency, the machine learning model was developed on a sampled subset of 2,000 examples; however, the statistical analysis used the entire dataset of 38,984 cases. This choice was made to ensure a more thorough and complete study of the relationships and distribution patterns between smoking status and clinical characteristics. Using the entire dataset for statistical evaluation improves statistical power, minimizes sampling bias, and catches more subtle trends across the population^32^.

The study tested the null hypothesis that there is no significant difference in health parameters between smokers and non-smokers. Independent T-tests were used for continuous variables (e.g., hemoglobin, cholesterol, blood pressure), assuming normality and homogeneity of variance, while Chi-square tests were applied to categorical attributes (e.g., dental caries, urine protein levels), assuming independence of observations. p-values were reported precisely (e.g., *p* = 0.025) to ensure statistical transparency^33^. Descriptive statistics summarized categorical variables using frequency distributions and proportions, whereas continuous variables were summarized using means, standard deviations, and interquartile ranges where applicable. Feature importance was assessed using mutual information, SHAP, and LIME, while pre-processing included normalization (Max Normalization) and handling outliers using the Interquartile Range (IQR) method^34^. The descriptive statistical measures of categorical attributes are shown in Table 4. From The descriptives, it can be seen that smokers have higher variability in urine protein and the presence of dental caries compared to non-smokers.

**Table 4:** Description and categories of categorical attributes in the Dataset.

	Smoking	*N*	Mode	SD	Minimum	Maximum
hearing(left)	Non-Smoker	24,666	1	0.165	1	2
	Smoker	14,318	1	0.143	1	2
hearing(right)	Non-Smoker	24,666	1	0.166	1	2
	Smoker	14,318	1	0.147	1	2
Urine protein	Non-Smoker	24,666	1	0.39	1	6
	Smoker	14,318	1	0.422	1	6
dental caries	Non-Smoker	24,666	0	0.385	0	1
	Smoker	14,318	0	0.445	0	1

**Fig. 2:**
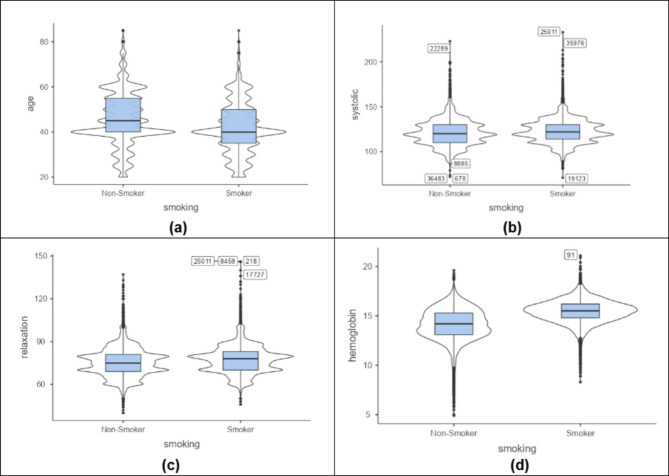
Violin Plots Showing Distribution of (**a**) Age, (**b**) Systolic Blood Pressure, (**c**) Diastolic Blood Pressure, and (**d**) Hemoglobin Levels.

**Fig. 3:**
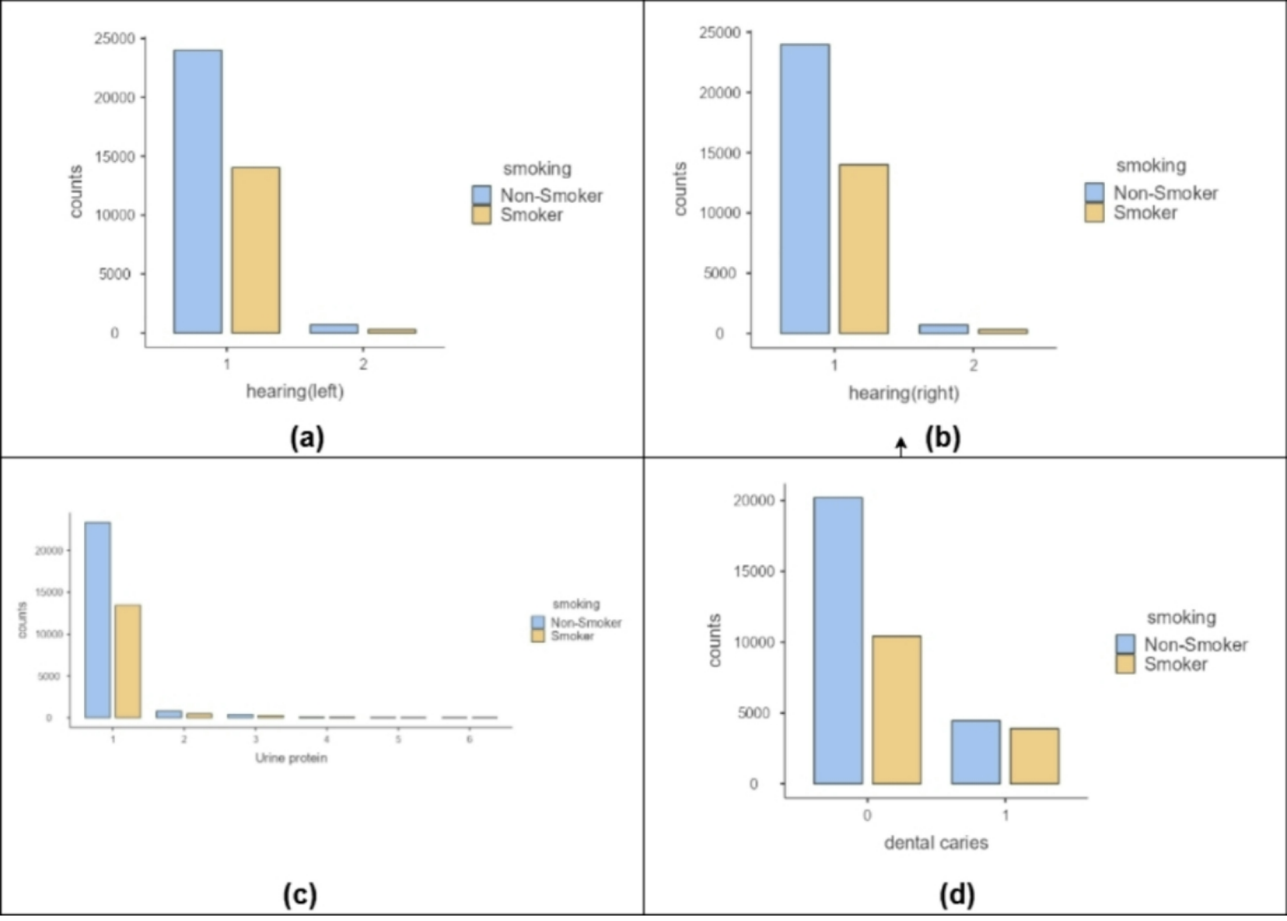
Multiple Bar Charts Depicting (**a**) Left Ear Hearing Status, (a) Right Ear Hearing Status, (**c**) Urine Protein Presence, and (**d**) Incidence of Dental Caries.

Violin charts that are described in Fig. 2 show that smoking is more common among younger individuals compared to older ones. Smokers tend to have higher blood pressure, indicating the impact of smoking. They also show more concentrated relaxation values than the broader range observed by non-smokers. Additionally, smokers have higher and more tightly clustered hemoglobin levels, while non-smokers have lower and more varied levels. The bar charts in Fig. 3 compare smokers and non-smokers across hearing, urine protein, and dental caries. Non-smokers have better hearing (normal category) and lower urine protein levels. However, smokers show a higher prevalence of dental caries.

**Fig. 4:**
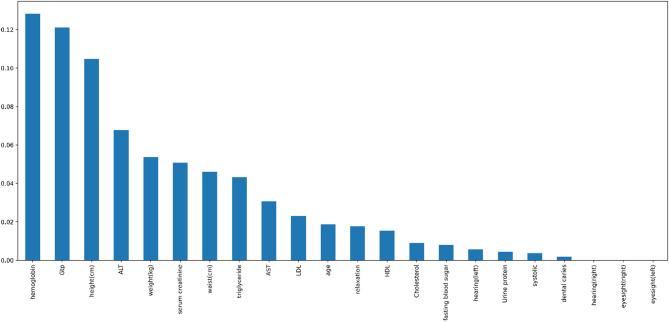
Feature Importance Ranked Using Mutual Information Scores.

The bar chart in Fig. 4 shows the mutual information between features and the target variable. Features such as “hemoglobin,” “GTP,” and “height(cm)” have the highest mutual information, indicating they are strongly associated with distinguishing smokers from non-smokers. On the other hand, features like “dental caries” and “eyesight(left)” have minimal mutual information, suggesting they contribute little to differentiating between the two groups. This analysis helps identify the most relevant features for predicting smoking behavior.

In statistical analysis, *p* < 0.001 means there is less than a 0.1% chance that the result happened by random chance. Table 5 presents T-test results showing statistically significant differences between the groups, suggesting a strong association between smoking status and the measured health variables. The chi-square test in Table 6 indicates a strong correlation between smoking status and categorical attributes, with dental caries showing the highest link. Effect sizes (Cohen’s d) were calculated for T-tests to quantify the magnitude of differences. Assumptions of normality and homogeneity of variances were checked for T-tests using Shapiro-Wilk and Levene’s tests respectively^35^. A significance threshold of α = 0.05 was used. For chi-square tests, independence of observations was assumed. Bonferroni correction was considered for multiple comparisons to control the family-wise error rate.

**Table 5:** Results of T-Test statistical Analysis.

Attributes	*P*	Cohen’s d
Age	< 0.001	**-0.36**
Height(cm)	< 0.001	**0.93**
Weight(kg)	< 0.001	**0.65**
Waist(cm)	< 0.001	**0.48**
Eyesight(left)	< 0.001	**0.13**
Eyesight(right)	< 0.001	**0.14**
Systolic Blood pressure	< 0.001	**0.15**
Relaxation Blood pressure	< 0.001	**0.22**
Fasting blood sugar	< 0.001	**0.20**
Cholesterol	< 0.001	**-0.06**
Triglyceride	< 0.001	**0.53**
HDL	< 0.001	**-0.39**
LDL	< 0.001	**-0.09**
Haemoglobin	< 0.001	**0.91**
Serum creatinine	< 0.001	**0.46**
AST	< 0.001	**0.13**
ALT	< 0.001	**0.20**
GTP	< 0.001	**0.46**

**Table 6:** Results of Chi-square (χ²) tests.

Attributes	Value	df	p
Hearing(left)	19	1	< 0.001
Hearing(right)	14.1	1	< 0.001
Urine Protein	11.1	5	< 0.001
Dental Carie	451	1	< 0.001

### C. Data preprocessing

A key step in preparing data for machine learning is through preprocessing. Because the dataset had no null values, dealing with missing data was unnecessary. Categorical features such as hearing and urine protein were label-encoded using sklearn’s LabelEncoder to convert them into numerical form suitable for ML models. To standardize, a max normalization method was used, in which each feature was scaled by dividing its values by the absolute maximum, ensuring that all features had comparable ranges between − 1 and 1, which enhances model performance. Max normalization was chosen to preserve relative feature scales and maintain interpretability, particularly because many features did not follow a normal distribution^36^. The dataset was already balanced, which meant that the classes in the target variable were equally represented, preventing class imbalance. No features were excluded, and no additional feature engineering was performed to preserve model interpretability. Finally, the data was ultimately split into training and testing sets in an 80 − 20 ratio, which is a conventional strategy for balancing training efficiency and evaluation dependability. A fixed random seed of 42 was used during stratified sampling and train-test splits to ensure reproducibility. An 80 − 20 split was applied with stratification to maintain class balance^37^. Since the sampled dataset was balanced (1,000 smokers and 1,000 non-smokers), the class distribution in the training and testing sets remained generally balanced, although little fluctuation may arise owing to randomization^38^.

In this study six ML models were employed, each with distinct learning principles. Logistic Regression models linear relationships^39^; Decision Trees and Random Forests create rule-based partitions^40^; KNN relies on instance proximity^41^; CatBoost uses gradient boosting to optimize accuracy on tabular data^42^; and ANN models complex patterns using layered neurons^43^. These methods were selected for their varied complexity, interpretability, and performance potential on clinical data. Metrics such as accuracy, precision, recall, F1 score, AUC, and confusion matrix are used to evaluate model performance alongside specific measures like Hamming Loss, Log Loss, Jaccard Score, and Matthews Correlation Coefficient. K-Fold Cross-Validation is utilized to ensure robust evaluation, splitting the data into multiple subsets for training and testing, with average scores providing a reliable assessment^44^. Ensemble stacking combines predictions from numerous base models (Random Forest, Logistic Regression, KNN, Decision Tree, and CatBoost) using Logistic Regression as a meta-classifier to increase overall prediction accuracy and dependability^45^. To prevent data leakage, the meta-learner in the stacked ensemble was trained on predictions made by the base models using a validation fold held out during the base model training^46^. This ensures the meta-learner does not see the same data twice.KNN2 in ensembling stacking represents a second configuration of the K-Nearest Neighbors model with different hyperparameters. The three search techniques, Grid Search, Randomized Search, and Bayesian Search, are used for hyperparameter optimization. Grid Search ensures a detailed exploration of all hyperparameter combinations, balancing its thoroughness with the high computational effort required^47^. Randomized Search samples a fixed number of random combinations, offering a faster alternative with reduced computational effort^48^.

**Fig. 5:**
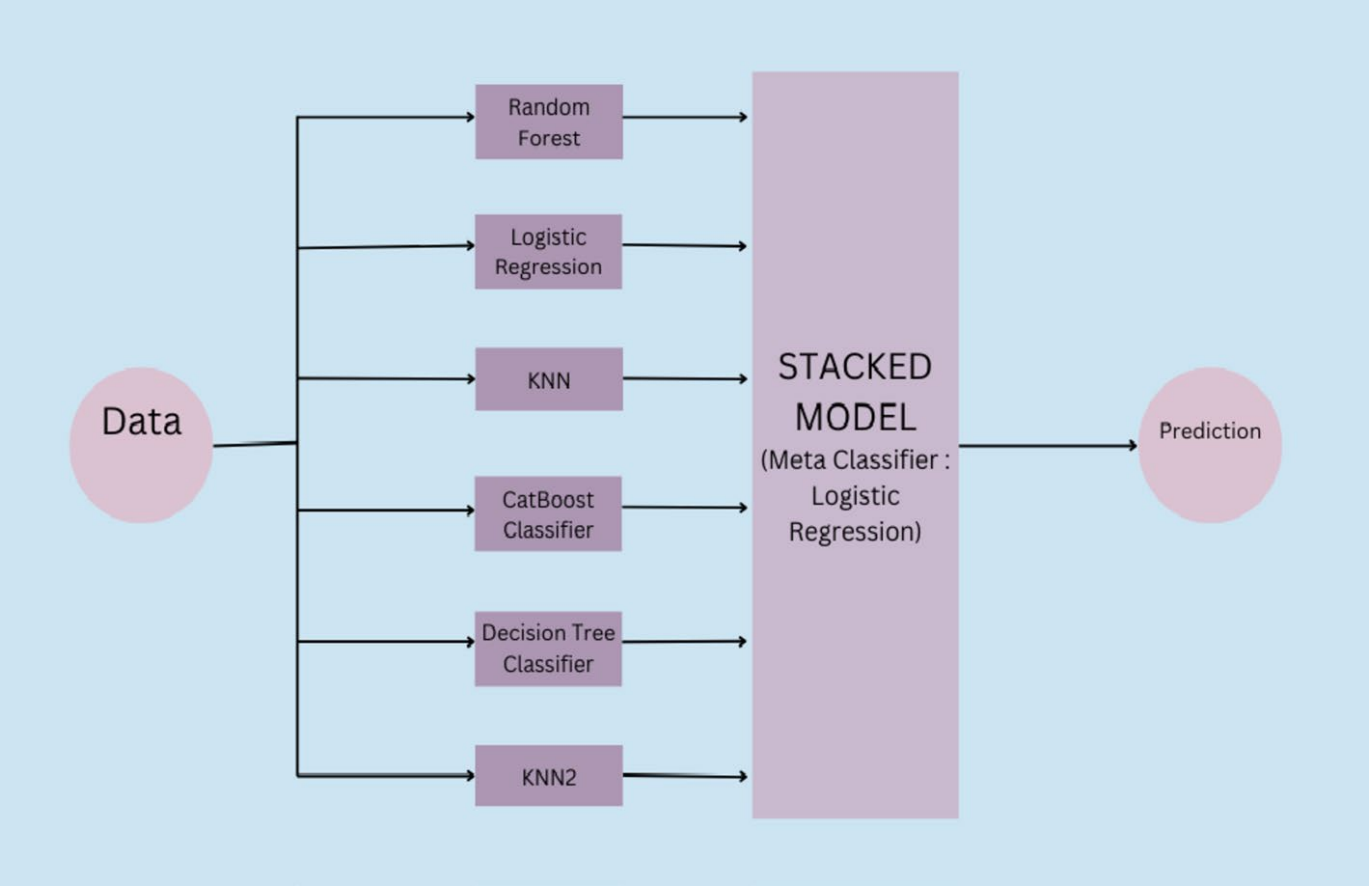
Architecture of the Proposed Stacked Machine Learning Model.

Bayesian Search iteratively selects hyperparameters based on prior evaluations, using probabilistic models to balance exploration and exploitation, making it more efficient for complex searches^49^. The custom stack used is described in Fig. 5.

Four XAI algorithms have also been used to make prediction interpretable. These techniques were employed after model training for interpretability purposes only, and not as a feature selection method prior to training.

SHAP: SHAP, rooted in game theory, is a robust tool for understanding machine learning model predictions. It assigns a SHAP value to each characteristic, indicating how it contributes to the prediction. SHAP guarantees consistency (unchanging impact for constant features) and fairness (equal contribution distribution). It is compatible with most algorithms and is available in Python. The technique entails calculating SHAP values, analyzing feature contributions, and displaying results using plots to improve comprehension^50^.

LIME: LIME is a tool introduced in 2016 that explains specific predictions of machine learning models. It modifies input variables, monitors forecast changes, and employs a basic linear model to approximate the complicated model locally. LIME is compatible with most algorithms, uses proximity-based weights, and presents findings in simple charts, making it ideal for clear, instance-level interpretations^51^.

QLattice: QLattice is a symbolic regression framework designed to handle both numerical and categorical data. It builds models based on QGraphs, which consist of nodes representing features, edges representing connections between nodes, and an activation function that modifies the output. This approach helps uncover simple, interpretable correlations within complex data^52^.

**Fig. 6:**
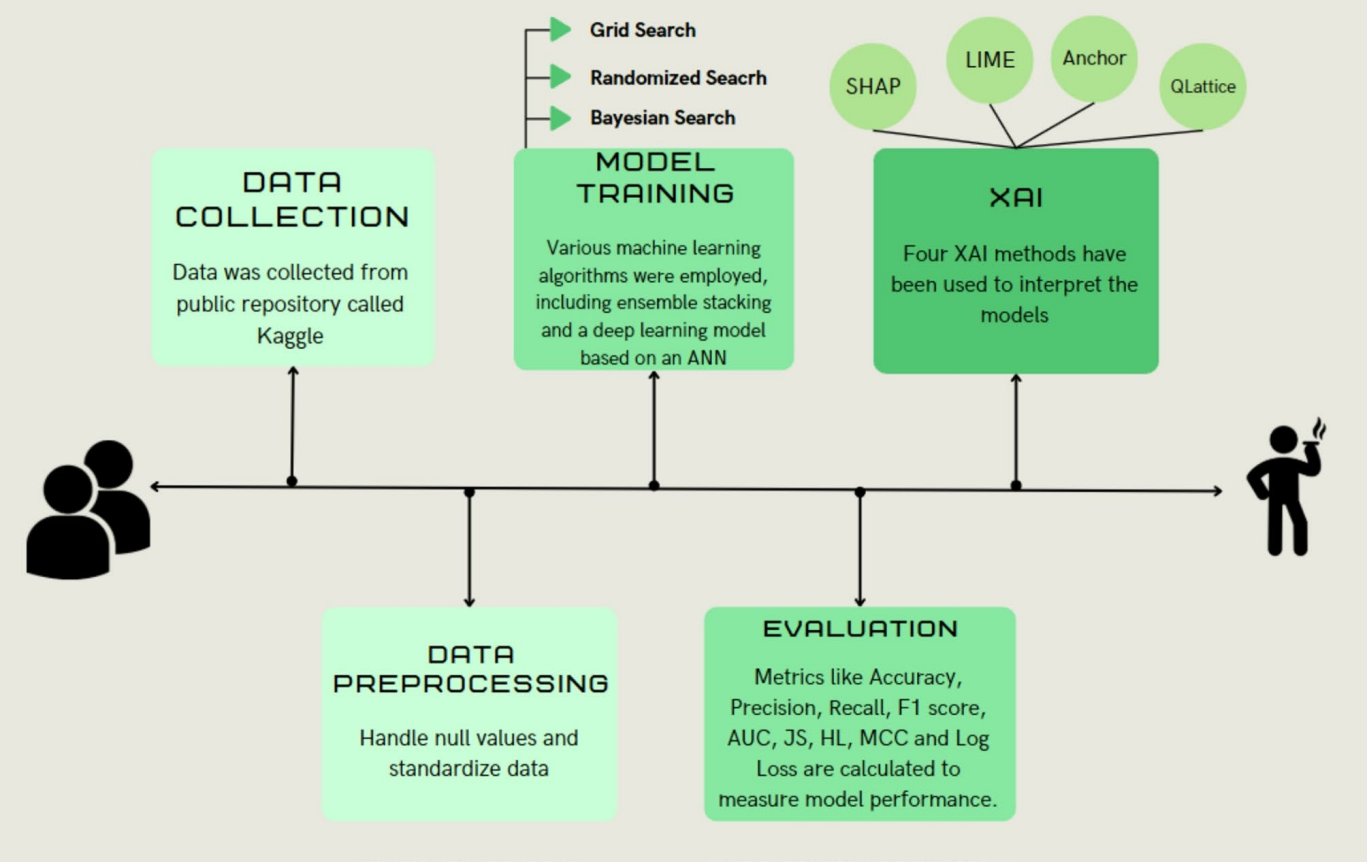
Workflow Diagram of the Machine Learning Process Implemented.

Anchor: Anchor is a model explanation strategy that employs “conditions” and “rules” to explain predictions. It discovers a group of features that result in consistent predictions when combined under conditions. The strength of an anchor is measured by two metrics: coverage, which indicates how many examples have the same condition, and precision, which shows the accuracy of the explanations^53^. Anchor provides precise and reliable local explanations for model predictions by focusing on these metrics. The machine learning process utilized is depicted in Fig. 6.

All model development and analysis were carried out using Python in the Google Colab environment which can be shared under data availability. For data preprocessing and manipulation, pandas (v1.5.3) and numpy (v1.24.2) were used. Visualizations were created using matplotlib (v3.7.1) and seaborn (v0.12.2). Machine learning models were implemented using scikit-learn (v1.2.2) and catboost (v1.2.2). The Artificial Neural Network was built using tensorflow (v2.12.0) and keras (v2.12.0). Model interpretability was enhanced using explainable AI libraries including SHAP (v0.41.0), LIME (v0.2.0.1), QLattice via feyn (v0.1.0), and Anchor (v0.0.3). Statistical analysis was performed using Jamovi (v2.6.0) and Python’s scipy (v1.10.1).

## Result

In this research, six classifiers were used to predict smoking status. Table 7 describes the performance metrics used to validate the classifier. Table 8 compares the performance metrics of various machine learning algorithms using three different hyperparameter search techniques: Grid Search, Randomized Search, and Bayesian Search. Random Forest under Grid Search achieved the higher accuracy of 0.8 and better overall metrics, highlighting its effectiveness among the evaluated combinations. To ensure robustness and reduce overfitting, five-fold cross-validation was employed during model training and evaluation. This approach allows averaging performance across multiple partitions of the dataset, leading to more generalizable results^54^.

**Table 7:** Performance metrics employed for classifier Validation.

Sl. No.	Metric name	Formula	Description
1	Accuracy	Accuracy = (TP + TN) / (TP + TN + FP + FN)	It measures how many correct predictions a machine learning model makes.
2	Precision	Precision = TP / (TP + FP)	It measures the number of positive classifications that were actually correct. Precision is high when false positives are low.
3	Recall	Recall = TP / (TP + FN)	It gauges how well the model can identify the positive class. Recall is high when false negatives are low.
4	F1-score	F1-score = 2 × (Precision × Recall) / (Precision + Recall)	A metric that combines both precision and recall.
5	AUC	-	In the receiver operating characteristic (ROC) curve, the true positive rate is plotted against the false positive rate at various thresholds. The area under this curve is called AUC (area under the curve).
6	Average Precision (AP)	-	In a precision-recall curve, precision is plotted against recall at various thresholds. The area under this curve is called average precision.
7	Jaccard Score (JS)	Jaccard Score = |A ∩ B| / |A ∪ B|	The degree of similarity between two groups of data is gauged by the Jaccard score.
8	Log Loss (LL)	Log Loss = - (1/N) ∑ [y_i log(p(y_i)) + (1 - y_i) log(1 - p(y_i))]	Measures how closely the predicted probability matches the true value.
9	Matthews Correlation Coefficient (MCC)	MCC = (TP × TN - FP × FN) / sqrt((TP + FP)(TP + FN)(TN + FP)(TN + FN))	Measures the difference between the actual and predicted values.

**Table 8:** Performance metrics of various machine learning algorithms Evaluated.

Algorithm	Accuracy	Precision	Recall	F1-Score	Area Under Curve	Hamming Loss	Jaccard Score	Log Loss	Mathews Correlation Coefficient
**Grid Search**									
Random Forest	0.8	0.8	0.80	0.79	0.84	0.205	0.672	7.389	0.5959
KNN	0.74	0.76	0.75	0.74	0.81	0.255	0.617	9,191	0.501
Decision Tree	0.66	0.68	0.66	0.65	0.71	0.345	0.5369	12.435	0.333
catBoost	0.78	0.78	0.78	0.77	0.84	0.225	0.643	8.109	0.554
Logistic Regression	0.74	0.75	0.75	0.74	0.84	0.255	0.605	9.191	0.494
Ensemble Stack	0.76	0.76	0.76	0.76	0.84	0.24	0.607	8.65	0.519
**Randomized Search**									
Random Forest	0.79	0.81	0.79	0.79	0.86	0.21	0.6865	7.5691	0.5945
KNN	0.72	0.73	0.72	0.72	0.8	0.2775	0.6007	10.0021	0.4519
Decision Tree	0.66	0.71	0.66	0.64	0.72	0.3375	0.5781	12.1647	0.3689
catBoost	0.78	0.79	0.78	0.77	0.83	0.225	0.6703	8.1098	0.5663
Logistic Regression	079	0.8	0.79	0.79	0.83	0.2125	0.6755	7.6592	0.5818
Ensemble Stack	0.76	0.77	0.76	0.76	0.83	0.24	0.6404	8.6504	0.5255
**Bayesian Optimization Search**									
Random Forest	0.77	0.79	0.77	0.77	0.84	0.2275	0.6654	8.1999	0.559
KNN	0.72	0.74	0.72	0.71	0.8	0.2825	0.6076	10.1823	0.4513
Decision Tree	0.74	0.75	0.74	0.73	0.8	0.2625	0.6196	0.8643	0.4836
catBoost	0.74	0.76	0.74	0.73	0.83	0.2625	0.66315	9.4614	0.4947
Logistic Regression	0.75	0.76	0.75	0.75	0.81	0.25	0.6282	9.0109	0.5052
Ensemble Stack	0.79	0.79	0.79	0.79	0.85	0.2125	0.6718	7.6592	0.5793
**Neural Network**									
ANN	0.74	0.75	0.74	0.74	-	-	-	-	-

Table 9 summarizes the hyperparameters selected for various machine learning algorithms after applying Grid Search, Bayesian Search, and Randomized Search. Hyperparameters are the adjustable settings of a model, such as the depth of a decision tree or the number of neighbors in KNN, which are set before training begins^55^. These values significantly influence the model’s performance and are optimized through different search techniques to find the best configuration for the task at hand. This ensures the models are fine-tuned for accuracy and efficiency^56^.

The hyperparameters for each model were selected using Grid Search, Randomized Search, and Bayesian Optimization based on validation performance. Parameters like n_estimators in Random Forest, n_neighbors in KNN, and learning_rate in CatBoost were tuned to balance accuracy and avoid overfitting^57^. The Logistic Regression model and custom stacking ensemble used standard regularization settings for simplicity and interpretability. Final values were chosen based on the best F1-score across five-fold cross-validation^58^.

**Table 9:** Hyperparameters chosen after utilizing various search techniques.

Algorithm	Grid Search	Bayesian Search	Randomized Search
Random Forest	{‘bootstrap’: True, ‘max_depth’: 80, ‘max_features’: 2, ‘min_samples_leaf’: 4, ‘min_samples_split’: 8, ‘n_estimators’: 100}	([(‘bootstrap’, True), (‘max_depth’, 110), (‘max_features’, 3), (‘min_samples_leaf’, 3), (‘min_samples_split’, 12), (‘n_estimators’, 100)])	{‘n_estimators’: 1000, ‘min_samples_split’: 8, ‘min_samples_leaf’: 4, ‘max_features’: 3, ‘max_depth’: 100, ‘bootstrap’: True}
KNN	{‘n_neighbors’: 35}	([(‘n_neighbors’, 51)])	{‘n_neighbors’: 60}
Decision Tree	{‘criterion’: ‘entropy’, ‘max_depth’: 5, ‘max_features’: ‘log2’, ‘min_samples_leaf’: 11, ‘min_samples_split’: 50, ‘splitter’: ‘best’}	([(‘criterion’, ‘entropy’), (‘max_depth’, 150), (‘max_features’, None), (‘min_samples_leaf’, 1), (‘min_samples_split’, 311), (‘splitter’, ‘best’)])	{‘splitter’: ‘best’, ‘min_samples_split’: 350, ‘min_samples_leaf’: 5, ‘max_features’: ‘sqrt’, ‘max_depth’: 15, ‘criterion’: ‘entropy’}
catBoost	{‘border_count’: 32, ‘depth’: 2, ‘iterations’: 250, ‘l2_leaf_reg’: 1, ‘learning_rate’: 0.03}	([(‘border_count’, 5), (‘depth’, 3), (‘iterations’, 100), (‘l2_leaf_reg’, 10), (‘learning_rate’, 0.03)])	{‘learning_rate’: 0.03, ‘l2_leaf_reg’: 1, ‘iterations’: 250, ‘depth’: 1, ‘border_count’: 10}
Logistic Regression	{‘C’: 100, ‘penalty’: ‘l2’}	([(‘C’, 100), (‘penalty’, ‘l2’)])	{‘penalty’: ‘l2’, ‘C’: 10}
Ensemble Stack	use_probas = True, average_probas = False, meta_classifier = logistic regression	use_probas = True, average_probas = False, meta_classifier = logistic regression	use_probas = True, average_probas = False, meta_classifier = logistic regression

**Fig. 7:**
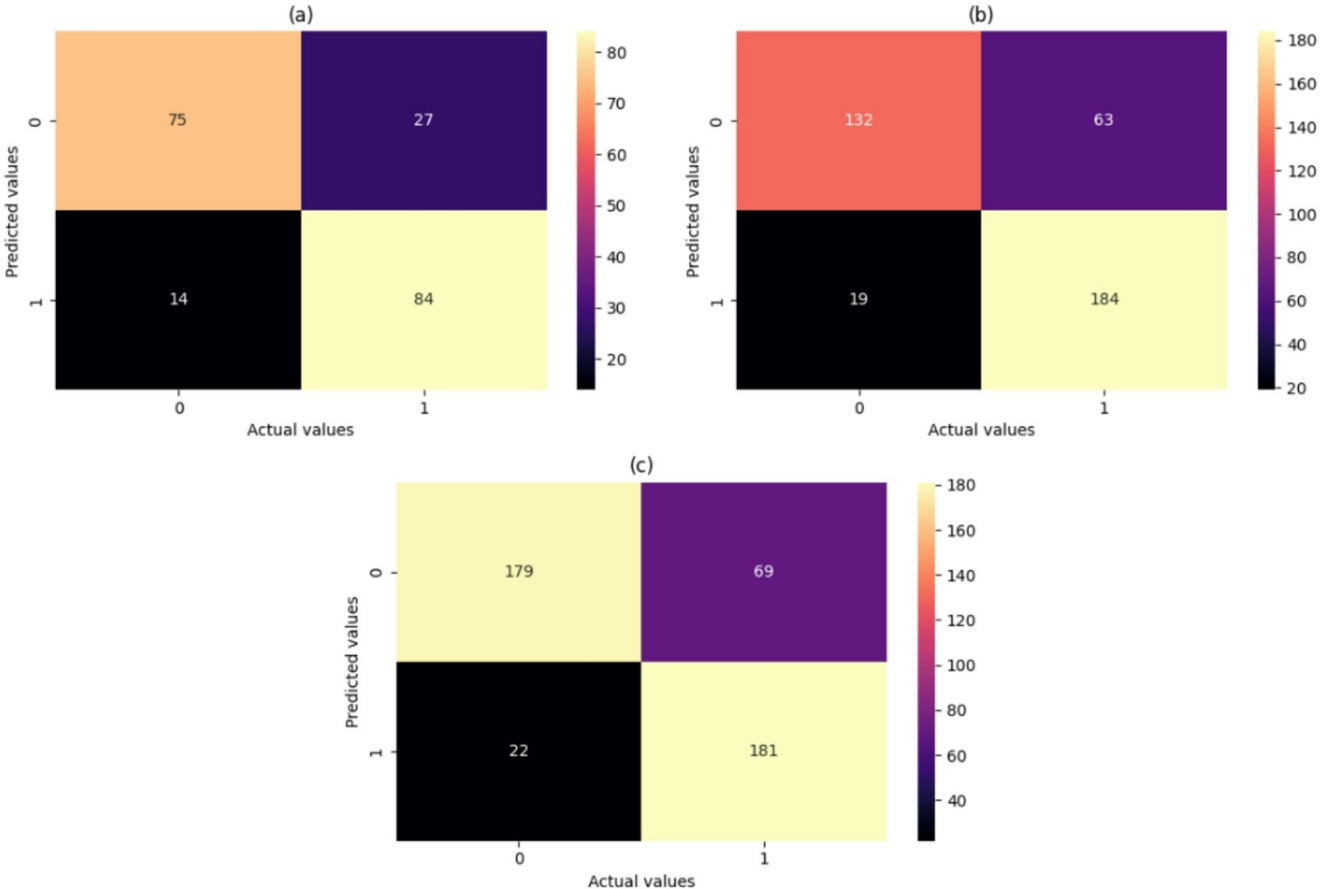
Confusion Matrices for Random Forest Classifier Using (a) Grid Search, (b) Randomized Search, and (c) Bayesian Optimization.

**Fig. 8:**
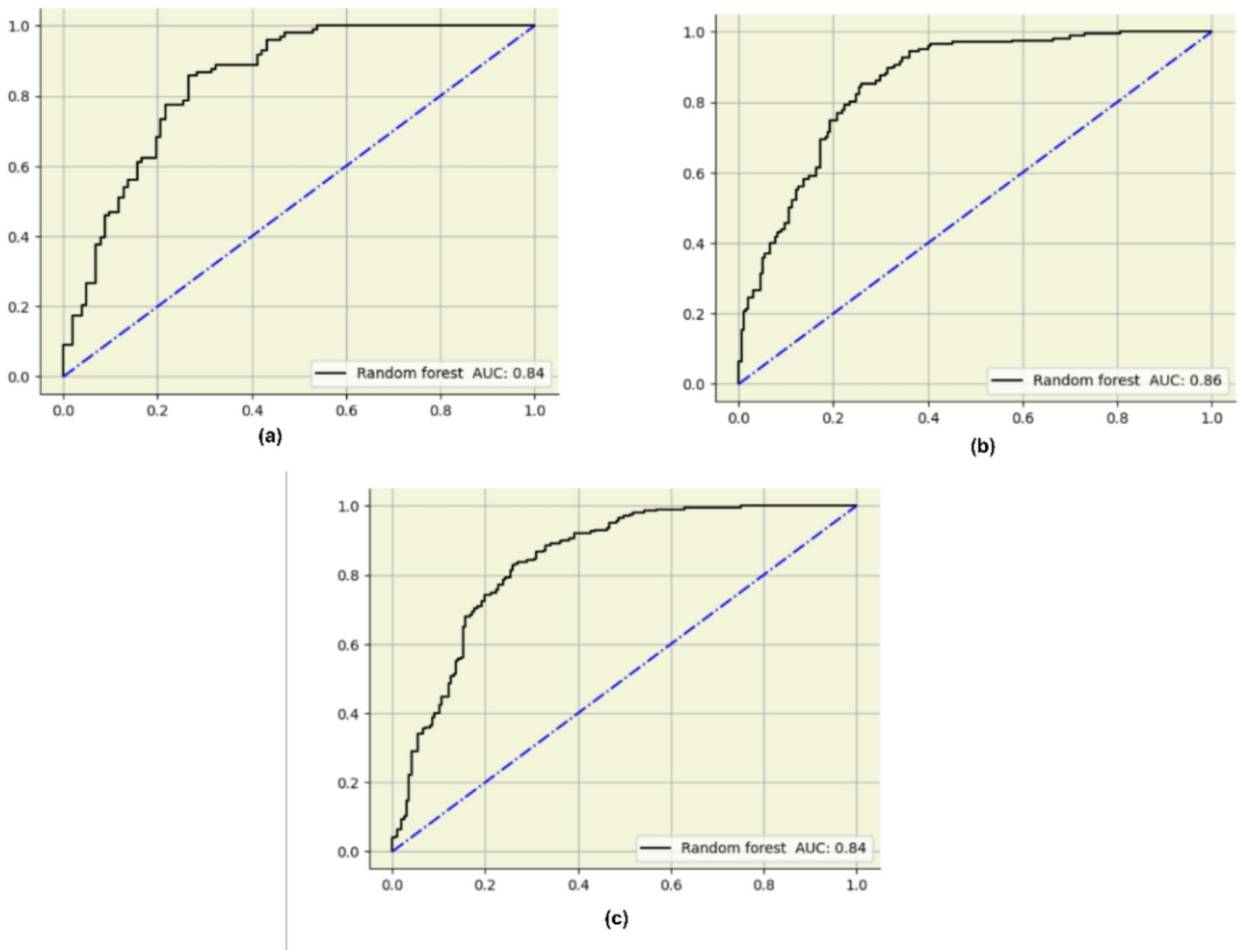
AUC Curves for Random Forest Classifier Using (a) Grid Search, (b) Randomized Search, and (c) Bayesian Optimization.

**Fig. 9:**
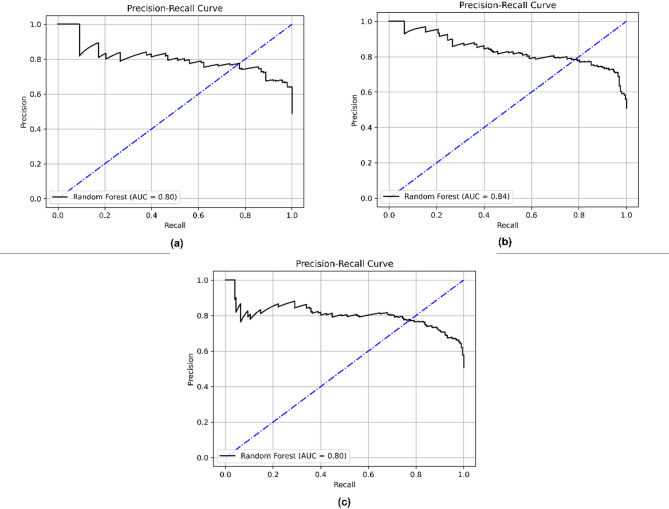
Precision-Recall Curves for Random Forest Classifier Using (a) Grid Search, (b) Randomized Search, and (c) Bayesian Optimization.

Although Random Forest achieved the bestter overall performance, other classifiers such as CatBoost and Logistic Regression also demonstrated competitive results. CatBoost achieved an F1-score of 0.77, while Logistic Regression reached 0.75. This supports the robustness of the classification task across diverse algorithms. The custom stack ensemble also yielded balanced performance with an F1-score of 0.76. Figure 7 is the confusion matrix for the Random Forest for the search method implemented. The confusion matrix reveals that Random Forest correctly predicted the majority of true negatives and true positives but misclassified a few false positives and false negatives. With fewer false positive and false negative results, the accuracy, precision, and recall were significantly higher. The AUCs of random forests are depicted in Fig. 8. The ROC curve for Random Forest illustrates its ability to classify the different classes accurately. With an AUC of 0.84 for GridSearch, 0.85 for Randomized search, and 0.84 for Bayesian search, the model performs strongly, outperforming the other models and suggesting better overall accuracy. The precision-recall Curve of the Random Forest in Fig. 9 shows how well the Random Forest model balances precision and recall at different thresholds. Four XAI models have been implemented to increase the interpretability of the result obtained by the model.

Aside from the machine learning methods discussed above, an Artificial Neural Network (ANN) was included to provide a comparison between tree-based models and a deep learning model, which is well-known for its effectiveness in health-related prediction tasks. Although not part of the ensemble, the ANN served as a benchmark to evaluate performance differences across algorithm families^59^. ANN is a computational model inspired by the structure of the human brain, using interconnected layers of neurons to recognize patterns and predict outcomes^60^. The architecture used in this study is detailed in Table 10.

**Fig. 10:**
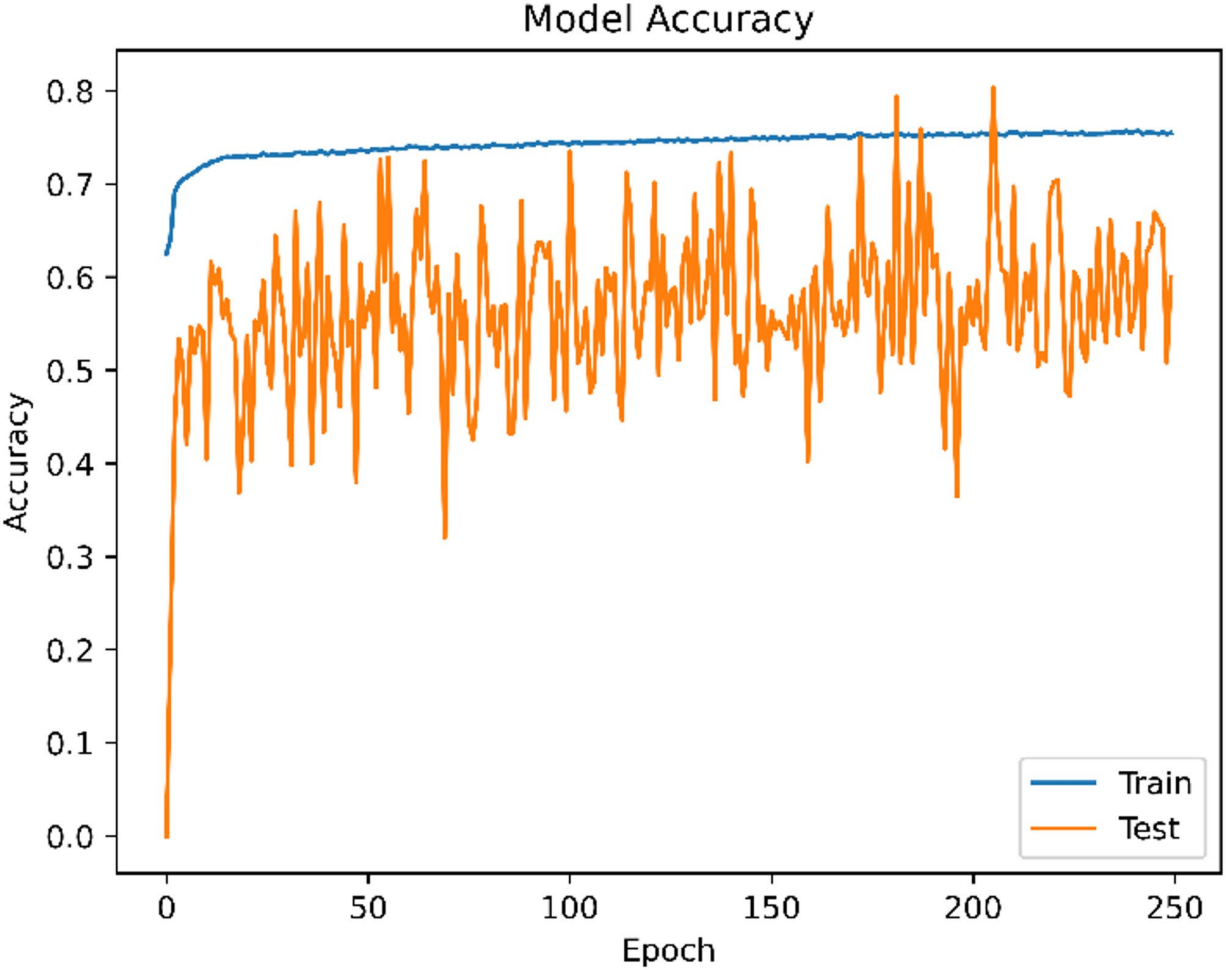
Accuracy Trend Over Training Epochs for the Artificial Neural Network Model.

**Fig. 11:**
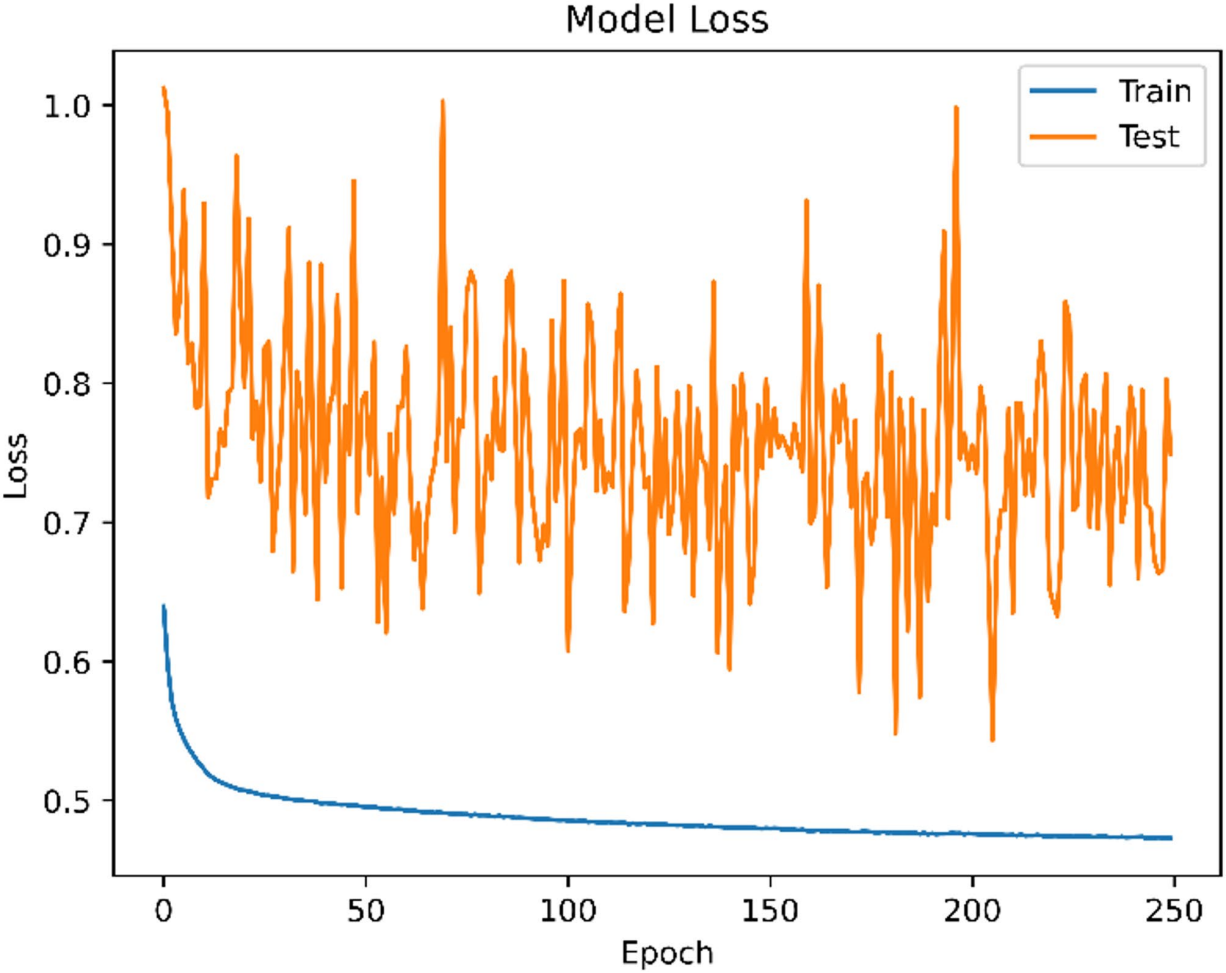
Loss Trend Over Training Epochs for the Artificial Neural Network Model.

The ANN model’s classification efficacy was assessed using common measures such as accuracy (0.74), precision (0.75), recall (0.74), and F1-score (0.74). The neural network consists of five layers, with neurons distributed as {30, 11, 7, 4, 1}. It was trained using the Adam optimizer (learning rate = 0.0001), binary cross-entropy loss, and ReLU and Sigmoid activation functions for the hidden and output layers, respectively. Training was conducted over 250 epochs with a batch size of 10 and a validation split of 0.2. No cross-validation was used for ANN; only the internal validation split was applied. Dropout layers and early stopping were deliberately excluded to maintain a simpler architecture for baseline evaluation. However, this decision introduced some overfitting, as evidenced in Figs. 10 and 11. The training loss decreased steadily and accuracy improved, but the validation accuracy and loss curves fluctuated considerably. This suggests that while the model learned well on training data, it exhibited inconsistent generalization on unseen data^61^. Future work will incorporate dropout regularization and early stopping mechanisms to stabilize performance and reduce overfitting.

**Table 10:** Model architecture of ANN Model.

Layer (type)	Output shape	Parameters
dense (Dense)	(None, 30)	690
dense_1 (Dense)	(None, 11)	341
dense_2 (Dense)	(None, 7)	84
dense_3 (Dense)	(None, 4)	32
dense_4 (Dense)	(None, 1)	5
Total parameters – 3,458		
Total trainable parameters − 1152		
Total non-trainable parameters − 0		

In this study, the Random Forest model beat the Artificial Neural Network (ANN), most likely because of the limited dataset and the applicability of tree-based models for tabular data. This comparison, however, is dataset-specific, and ANN performance may improve as datasets grow in size and complexity.

**Fig. 12:**
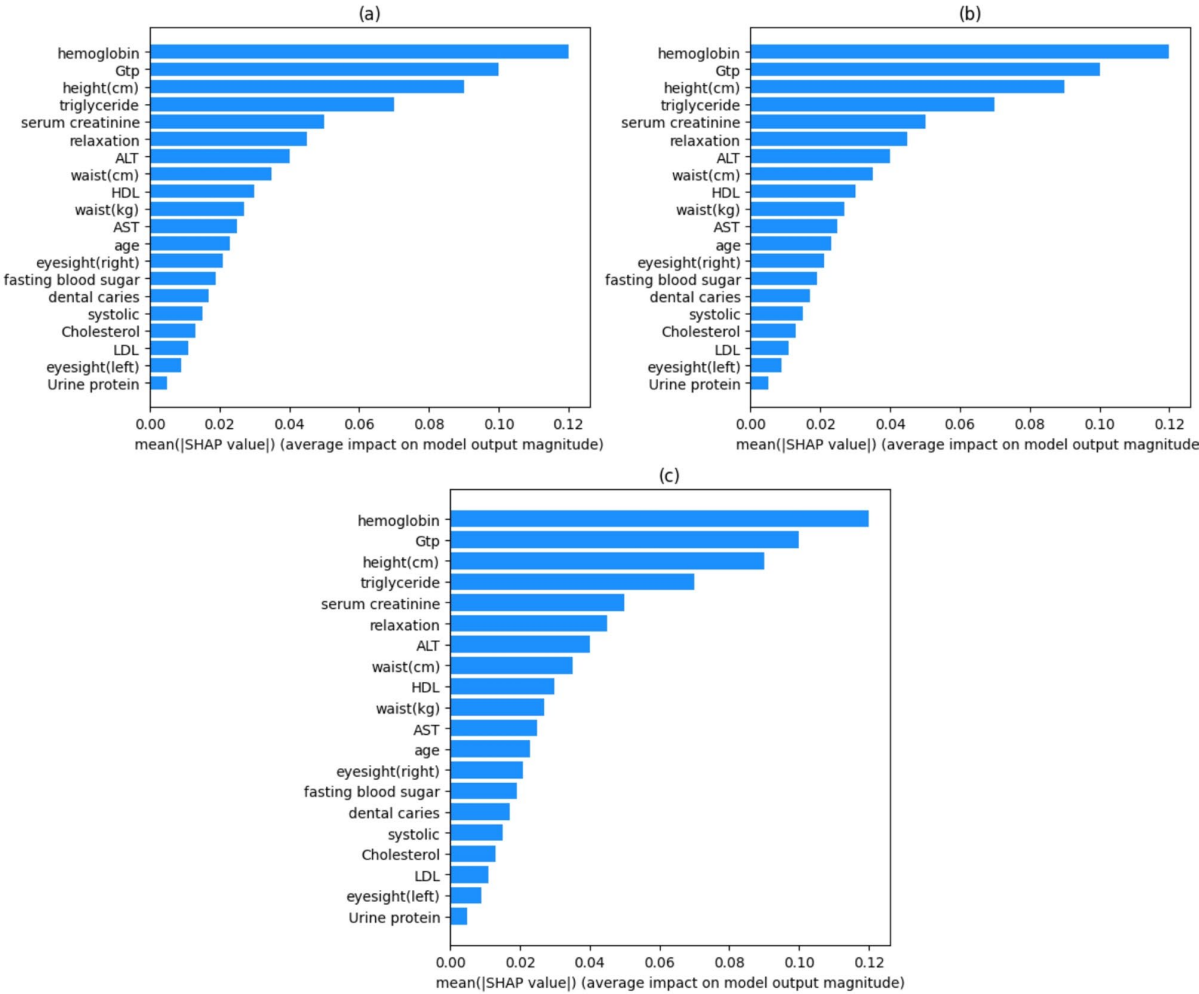
SHAP Mean Bar Plots Illustrating Model Interpretation for (a) Grid Search, (b) Randomized Search, and (c) Bayesian Optimization.

**Fig. 13:**
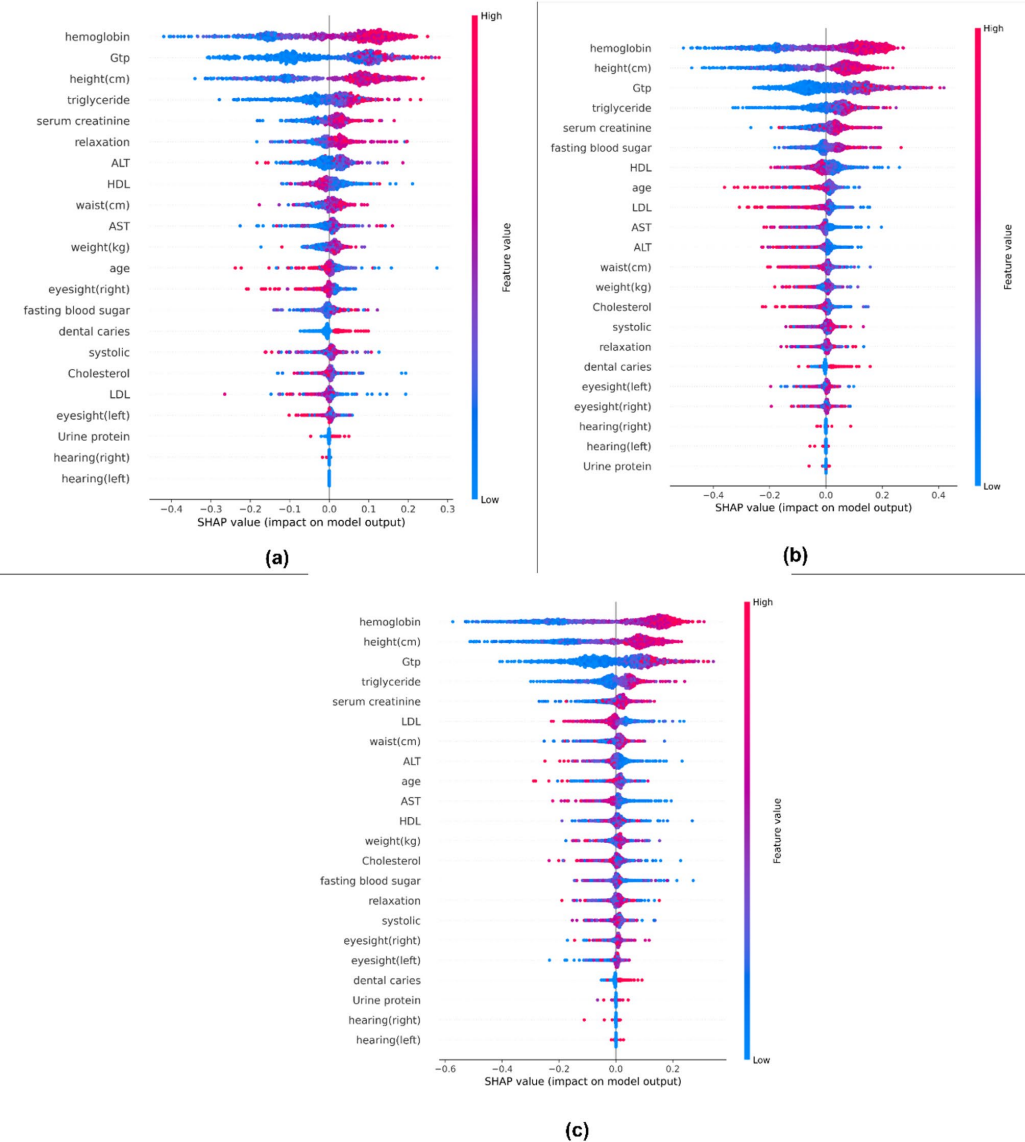
SHAP Beeswarm Plots for Model Interpretation Across (a) Grid Search, (b) Randomized Search, and (c) Bayesian Optimization.

Figures 12 and 13 employ SHAP values to demonstrate how various health factors influence a machine learning model’s predictions. Figure 12, a bar chart of average SHAP values, focuses on hemoglobin, GTP, and height as the most vital features. Figure 13 depicts a SHAP value distribution plot, with feature values color-coded (blue for low, red for high) and their position on the x-axis indicating whether they positively or adversely affect predictions. Key patterns emerge: hemoglobin and GTP have a strong influence that varies with their values, while features like hearing tests have minimal impact. Others, such as triglyceride and serum creatinine, show distinct clusters, reflecting their variable implications. This analysis helps medical professionals prioritize the most critical health parameters for better decision-making^62^.

**Fig. 14:**
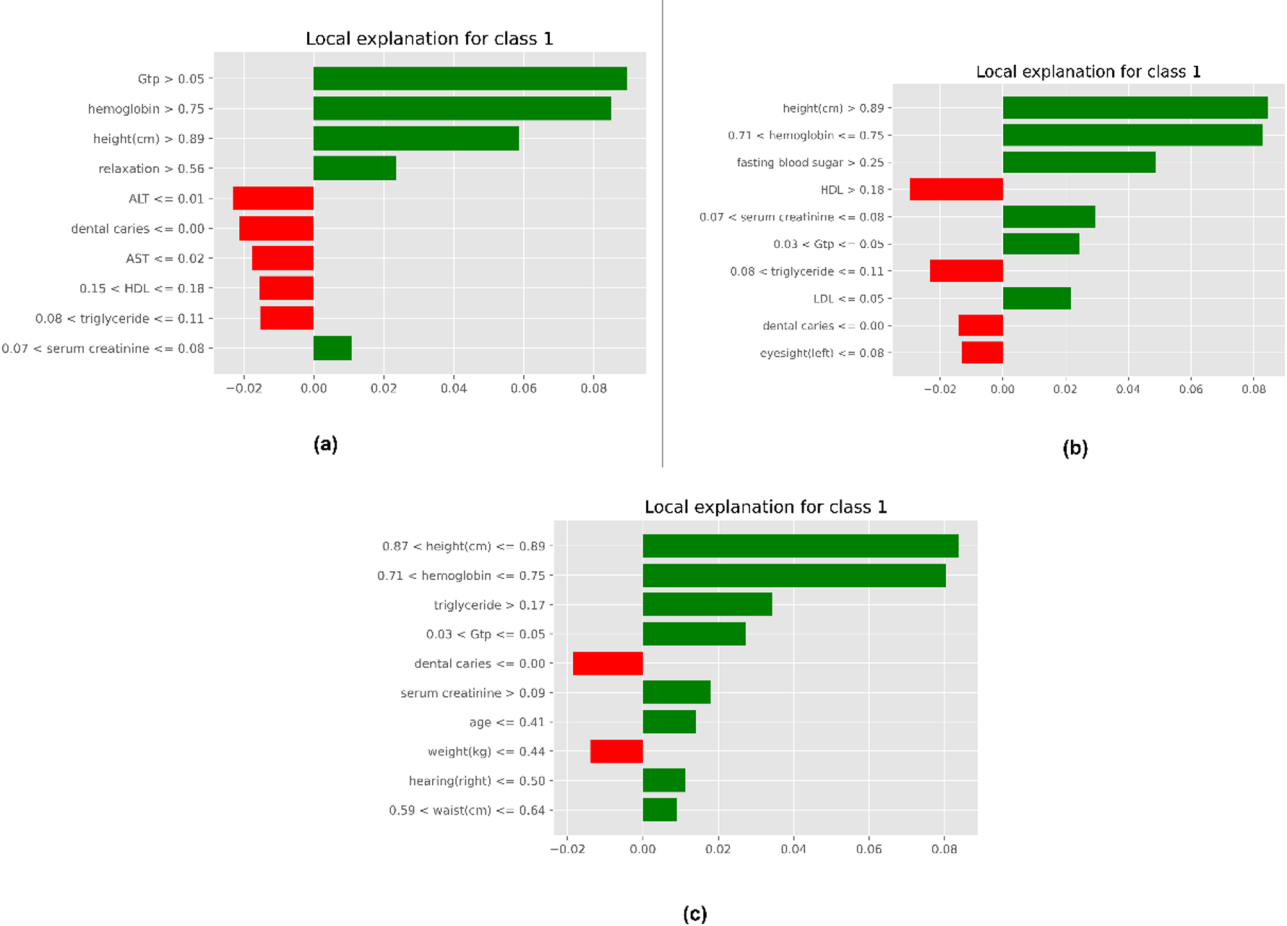
LIME-Based Feature Importance Visualizations for Models Trained Using (a) Grid Search, (b) Randomized Search, and (c) Bayesian Optimization.

Figure 14 illustrates local explanations for class 1 predictions for three search techniques, showcasing the impact of various features on the classification decisions. Positive contributions to the prediction are shown in green, while negative contributions are depicted in red. Across all techniques, features such.

as height, hemoglobin, and other physiological attributes consistently demonstrate a strong positive influence on class 1 predictions. Conversely, features like dental caries, serum creatinine, and specific lipid metrics are identified as negative contributors.

**Fig. 15:**
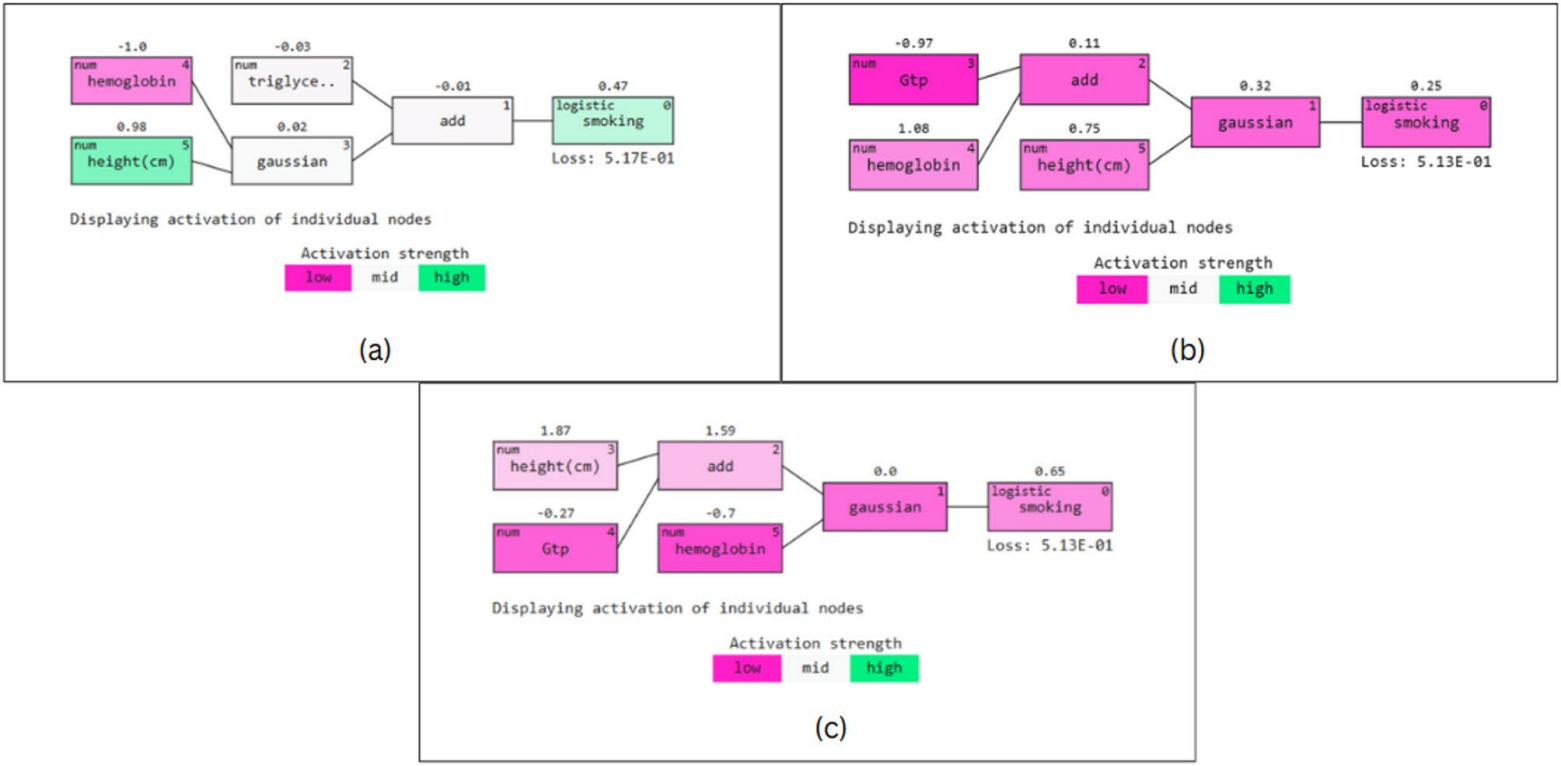
QGraphs Depicting Important Predictive Markers Identified by Models Using (a) Grid Search, (b) Randomized Search, and (c) Bayesian Optimization.

In Fig. 15, the three panels illustrate models generated by QLattice, showcasing the contributions of various features to predict smoking status. QLattice, a symbolic regression-based framework, generates mathematical models that express relationships between features. In our case, it produced rules like ‘smoking = GTP × height - hemoglobin,’ identifying meaningful interactions that contributed to classification. The strength of QLattice lies in its ability to discover compact, interpretable expressions that reveal non-linear relationships.

Across all panels, height (cm) consistently demonstrates a strong positive influence, with values like 0.98 in (a) and 1.59 in (c), making it a key predictor. Hemoglobin, on the other hand, exhibits a significant but variable contribution, being negative (-1.0) in (a) and (c), indicating its complex role in the prediction. Additional attributes such as triglycerides, Gaussian transformations, and GTP make smaller contributions, enhancing the model’s interpretability.

Anchors are interpretable decision rules that explain model predictions by emphasizing essential criteria that result in specific classifications. Anchor explains individual predictions using IF-THEN rule-based conditions with associated precision and coverage. For example, the rule ‘IF GTP > 0.05 AND hemoglobin > 0.71 THEN smoker’ had a precision of 0.89 and coverage of 0.17, making the explanations both interpretable and locally faithful. It is found from Table 11 that the most relevant qualities are hemoglobin and GTP, which emerge consistently across several criteria for both smokers and nonsmokers. Other factors, including height, weight, and cholesterol, play a role but are not as important. These rules provide unambiguous, human-readable insights into the model’s decision-making.

**Table 11:** Explanations generated by anchor explainer for predicting smoking Status.

Technique	Class	Anchor Rule	Precision	Coverage
Grid Search	Not Smoker	hemoglobin ≤ 0.65 AND GTP ≤ 0.02	0.95	0.15
		GTP > 0.05 AND Cholesterol ≤ 0.39	0.75	0.06
		hemoglobin > 0.71 AND height(cm) > 0.87	0.71	0.33
		GTP > 0.02 AND hemoglobin > 0.71	0.75	0.43
	Smoker	GTP > 0.05 AND hemoglobin > 0.71	0.89	0.17
		GTP ≤ 0.03 AND weight(kg) ≤ 0.48	0.69	0.37
		hemoglobin ≤ 0.65 AND height(cm) ≤ 0.84	0.90	0.21
		GTP ≤ 0.02 AND ALT ≤ 0.01	0.81	0.15
Randomized Search	Not Smoker	GTP ≤ 0.02 AND hemoglobin ≤ 0.65	0.92	0.12
		GTP ≤ 0.02 AND height(cm) ≤ 0.84	0.89	0.14
		hemoglobin ≤ 0.71 AND serum creatinine ≤ 0.07	0.74	0.31
		GTP ≤ 0.03 AND height(cm) ≤ 0.84	0.86	0.23
	Smoker	GTP > 0.03 AND height(cm) > 0.87	0.79	0.27–0.28
		GTP > 0.05 AND age ≤ 0.41	0.85	0.07
		height(cm) > 0.89 AND hemoglobin > 0.71	0.72	0.17
Bayesian Optimization	Not Smoker	LDL ≤ 0.06 AND height(cm) > 0.87	0.75	0.25
		GTP ≤ 0.02 AND hemoglobin ≤ 0.71	0.79	0.21
		hemoglobin ≤ 0.65 AND height(cm) ≤ 0.84	0.93	0.20
		hemoglobin ≤ 0.65 AND weight(kg) ≤ 0.44	0.83	0.19
		hemoglobin > 0.71 AND height(cm) > 0.84	0.71	0.43
	Smoker	GTP > 0.03 AND height(cm) > 0.87	0.79	0.29
		hemoglobin > 0.71 AND height(cm) > 0.87	0.71	0.33
		GTP ≤ 0.03 AND triglyceride ≤ 0.11	0.70	0.35
		GTP > 0.03 AND hemoglobin > 0.71	0.76	0.31

Feature interactions were inferred from SHAP plots and QLattice visualizations, where combinations such as hemoglobin-GTP and height appeared repeatedly in predictive rule sets. These interactions were further supported by their high mutual information and joint presence in multiple XAI explanations.Hemoglobin and GTP levels have known associations with smoking. Smoking can cause elevated GTP due to liver stress and inflammation, and it may affect hemoglobin levels through increased carbon monoxide exposure, leading to compensatory erythropoiesis. These physiological changes explain why these features emerged as strong predictors in our models^63^.

The important attributes identified by different XAI models and statistical interpreters are depicted in Table 12.

**Table 12:** Key attributes identified by different explainable AI (XAI) techniques and Interpreters.

Method	Key Attributes Identified
Mutual Information	Hemoglobin, GTP, height, ALT, Weight
SHAP	Hemoglobin, GTP, height, triglycerides, Serum creatinine.
LIME	GTP, hemoglobin, blood pressure, triglycerides, Serum creatinine.
QLattice	Hemoglobin, height, triglycerides, GTP, Serum creatinine.
Anchor	Hemoglobin, GTP, height, weight, cholesterol.

## Discussion

This study implemented multiple machine learning classifiers and ANN to predict smoking status based on clinical bio signal parameters. Three different search techniques were also implemented on different AI models. Among the six classifiers tested, the Random Forest model performed the best, achieving an accuracy of 80% for the grid search technique, 79% for the randomized search, and 77% for the Bayesian search technique, along with high precision, recall, and F1 scores. Four XAI techniques, SHAP, LIME, QLattice, and Anchor, were employed to improve transparency and interpretability. These methods identified hemoglobin, GTP, and height as the most influential features in predicting smoking status. Our study not only achieved competitive accuracy but also introduced a broader range of explainability techniques compared to existing research, enhancing both the interpretability and medical applicability of the model. In comparison, other studies either do not use explainability methods or rely on fewer tools, making our approach more comprehensive. The interpretability of this model enables healthcare professionals to understand and trust its outputs, potentially guiding patient-specific preventive strategies. Our findings align with and improve upon previous studies in smoking prediction. For example, Ammar et al.^11^ achieved 77% accuracy using an ensemble deep learning approach, while our Random Forest model achieved an accuracy of 80% with better interpretability. Additionally, prior studies like^20^ incorporated SHAP and LIME, but did not explore multiple XAI methods together. Table 13 summarizes performance comparisons with related works. The results revealed essential trends in smokers and non-smokers. Hemoglobin levels were notably higher in smokers, reflecting disruptions in oxygen transport that are often associated with smoking^64^. GTP levels were also elevated among smokers, indicating potential liver stress^65^. Blood pressure, particularly systolic and diastolic readings, was elevated in smokers, highlighting cardiovascular risks^66^. Additionally, smokers exhibited higher fasting blood sugar levels, potentially signaling metabolic imbalances^67^. Dental caries were more prevalent in smokers, reinforcing the adverse impact of smoking on oral health^68^. Elevated triglycerides in smokers further emphasize their increased susceptibility to cardiovascular conditions^69^. These attributes were accurately identified by the classifiers and XAI techniques, ensuring precise and interpretable predictions that can aid healthcare professionals.

**Table 13:** Comparison of model performance and explainability techniques in related Studies.

Reference	Result	Explainers Used
^16^	80% accuracy	-
^17^	96.29%. F1-score	-
^18^	84.73% accuracy	-
^19^	83.29% accuracy	-
^20^	79.65% accuracy	SHAP, LIME
Our Study	80% accuracy	SHAP, LIME, Qlattice, Anchor

Despite the positive outcomes, this study had some drawbacks. It solely employed the clinical variables provided and excluded aspects such as alcohol usage or physical activity, which could improve the model’s predictions. Furthermore, the dataset was static and did not account for changes in health over time, making it less adaptive to changing circumstances. This study focused on predictive performance and interpretability using clinical features. Although age was included in the analysis, we did not perform subgroup analysis to evaluate whether the model’s predictions vary across different age ranges or populations. Ensuring fairness and avoiding bias in medical AI is essential. Therefore, future work will focus on fairness-aware techniques and subgroup evaluations to assess whether predictions remain consistent across different demographic or clinical groups.While performance metrics were validated using cross-validation, confidence intervals for these estimates were not computed in the current study due to resource constraints. Incorporating confidence intervals in future work would enhance the statistical robustness and interpretability of the results^70^.While the highest accuracy achieved was 0.80, this may be considered modest for high-stakes clinical settings. Future improvements could focus on increasing the model’s scalability and real-world applicability. Exploring deep learning models may increase performance, especially when using larger datasets, by revealing intricate patterns in the data^71^. Furthermore, employing cloud-based tools would improve the model’s accessibility and allow for more straightforward updating with fresh data, assuring its relevance over time. Deploying the model in real-world contexts such as schools, workplaces, or health programs may assist in refining its predictions and demonstrate its efficacy across varied populations.

Compared to prior studies in this domain, our approach offers a unique integration of multiple XAI techniques, enabling both global and local interpretability. Unlike traditional black-box models, our framework reveals clinically aligned interactions—such as between hemoglobin, GTP, and height—while maintaining robust predictive performance. This positions our model as both transparent and practically informative, enhancing its potential for adoption in real-world healthcare settings.

## Conclusion

This study showed that machine learning and deep learning algorithms can accurately predict smoking status, with Random Forest outperforming other models. The study used XAI algorithms to identify critical health markers such as hemoglobin and GTP, providing interpretable insights regarding smoking-related health patterns. These models have primary practical uses, such as assisting healthcare practitioners with early detection, enabling timely medical treatments, and personalizing treatment programs for people depending on their health profiles. Beyond clinical settings, the approach can help parents and guardians recognize smoking patterns in children and adolescents, allowing for early intervention and preventive interventions. Furthermore, these technologies can help institutions that perform health screenings, such as schools, workplaces, or community health initiatives, address smoking-related concerns more proactively. These models can also be used by public health authorities to create targeted awareness programs and identify populations that are more likely to develop smoking-related ailments. This model could be implemented into electronic health record (EHR) systems to serve as a decision support tool for identifying persons at risk of smoking-related problems. Its use of conventional clinical lab results qualifies it for routine screenings in occupational health, primary care, and preventive health programs. Future studies can assess adoption in real-world healthcare workflows. With its capacity to lessen reliance on self-reported data and give objective assessments, this technique represents a promising step forward in addressing the global smoking pandemic and encouraging improved health outcomes across all sectors.

## Data Availability

The datasets used and/or analysed during the current study available from the corresponding author on reasonable request.
